# The stable brother hiding in the shadow—news on intermediate filaments

**DOI:** 10.1007/s00709-020-01541-w

**Published:** 2020-08-17

**Authors:** Peter Nick

**Affiliations:** Botanical Institute, Karlsruher Institut für Technologie, Karlsruhe, Germany

The term “cytoskeleton” has been coined, when, from the early 1960s, electron microscopy has revealed filamentous structures that seemed to organise the cytoplasm (“microtubules”, Ledbetter and Porter 1963; “microfilaments”, Kamiya and Kuroda 1966). Only with the advent of fluorescence microscopy, it was recognised that this skeleton rather represents a very dynamic network, where both microtubules and actin filaments are continuously and rapidly turning over. In contrast, intermediate filaments, the often overlooked third element of the cytoskeleton, are actually closest to that what one could call a “skeleton”. They are described as stable, structural elements that not only maintain the intracellular architecture, for instance by keeping the nucleus in shape, but also sometimes even structure entire tissues, such as skins, feathers or corneous structures. In the walled cells of fungi and plants, this architectural role has been adopted by the cell wall, and this might be the reason, why intermediate filaments apparently were lost during the evolution of these life forms. However, since this third component of the filamentous cytoskeleton has never enjoyed the limelight of scientific attention, it has remained a bit of a terra incognita lurking in the shadow. That the intermediate filaments are merely a stable structure that, once generated, just persists, and is absent from walled plant cells, might be more a kind of dogma rooted in our ignorance, rather than a scientific fact. Two contributions in the current issue, one from the animal and one from the plant field, suggest that we should start to critically question this dogma.

The first contribution by Alibardi (2020b) in the current issue investigates the role of specific keratins during the formation of the caruncle of turtles, a juvenile structure on the beak that helps the young animal to hatch from the egg and, therefore, is also referred to as “egg tooth”. Like birds and the primitive mammal *Platypus*, turtles have replaced their teeth by a corneous layer, a developmental change that is linked with a loss of function for sonic hedgehog, a signalling protein required for dentition. The adult beak of turtles is then formed by so called β-keratins, intermediate filament proteins that differ from the canonical keratins (Alibardi 2020a). While it is clear that the egg-tooth must be composed of keratinous material, because it has to be hard enough to break the egg shell, the molecular composition, and its genesis as well as its transient existence (the egg tooth is shed a few days after hatching, in contrast to the beak), is unknown. Using specific antibodies against intermediate filament keratins, and two turtle-specific β-keratins, the author follows the histological differentiation of the turtle caruncle. He can demonstrate how epidermal cells that are subtended by maxilla and mandibles proliferate and form placodes that subsequently fuse into the beak, kept together by gap junctions, and forming a corneous epithelium accumulating intermediate filament keratins, complemented later β-keratins to form the corneous beak. This programme is then recapitulated in a constrained region of the corneous layer, starting from local proliferation, accumulation of intermediate filament keratins at the surface, and accumulation of β-keratins in the subtending, large so-called beta cells that are joined by adhesion proteins, such that a hard and stiff structure is formed. This caruncle can then later, once it has fulfilled its function, be shed altogether.

The contribution by Utsunomiya et al. (2020) adds a new facet to a controversial debate that had started already in the 1980s: Do plant cells possess intermediate filaments? Since structural support can be maintained by tethering to the solid cell wall, intermediate filaments might be obsolete. Moreover, plant nuclei seem to lack the nuclear lamina that keeps animal nuclei in shape. Nevertheless, antibodies raised against animal intermediate filaments were repeatedly reported to detect fibrillary structures in plant cells that were adjacent to microtubules (Fairbairn et al. 1994; Mizuno 1995), and soluble proteins purified from various plants were able to assemble filamentous structures of around 10 nm in diameter what would qualify them as intermediate filament proteins (for instance Mizuno 1995). However, when the first plant genomes became available, the search for canonical homologues of animal intermediate filaments was not very successful, such that the issue shifted a bit out of focus. The authors revisited this debate using a new approach—while canonical homologues to animal intermediate filaments seem to be absent from plants, still modular elements of these proteins might have been retained. Using this heuristic strategy, the authors could identify several candidates from the *Arabidopsis* genome. These candidates were, subsequently, fused to GFP and transformed into tobacco BY-2 cells. In fact, for one of these candidates, later termed as Intermediate Filament Motif Protein 1, with a weak (around 20%) similarity to lamin B, cytoskeletal patterns were observed, such that the authors subjected this protein to higher scrutiny. Using synchronised tobacco cells, this protein was found in mitotic microtubule structures, such as spindles and phragmoplast. Also during interphase, fibrillar structures were detected that were independent of microtubules and associated with the moving nucleus, reminiscent of actin rings that had been reported for migrating nuclei in the giant internodes of Characean algae (Wasteneys and Williamson 1991), or tobacco cells expressing a tetrameric fluorescent protein in fusion with the actin-binding Lifeact motif (Durst et al. 2014). Thus, the debate about plant intermediate filaments has been re-opened—due to the strong sequence divergence, this and possibly other plant homologues might have been simply overlooked.

Both contributions not only extend our (admittedly limited) knowledge about intermediate filaments but they also show that these protein structures harbour both developmental and cell-cycle-dependent dynamics and seem to be more than just a static “skeleton”. They also show that we have to be careful to transfer textbook statements from mammalian models to other life forms, especially for this still enigmatic and hardly investigated part of the cytoskeleton.
